# Regular exercise and the trajectory of health-related quality of life among Taiwanese adults: a cohort study analysis 2006–2014

**DOI:** 10.1186/s12889-019-7662-8

**Published:** 2019-10-23

**Authors:** Huan-Cheng Chang, Jersey Liang, Hui-Chuan Hsu, Sung-Kai Lin, Ting-Huan Chang, Shu-Hui Liu

**Affiliations:** 1 Division of Family Medicine, Department of Community Medicine, Landseed International Hospital, No.77, Guangtai Rd., Pingzhen Dist., Taoyuan, 32449 Taiwan, Republic of China; 2 Department of Health Care Management, Chang Gung University, No.259, Wen-Hwa 1st Rd., Kwei-Shan Dist., Taoyuan, 33302 Taiwan, Republic of China; 3 Department of Health Management and Policy, School of Public Health, University of Michigan, 1415 Washington Heights, M3007, SPH II, Ann Arbor, MI 48109 USA; 4 School of Public Health, Taipei Medical University, No.250, Wuxing Street, Taipei, 11031 Taiwan, Republic of China; 5 Research Center of Health Equity, College of Public Health, Taipei Medical University, No.250, Wuxing Street, Taipei, 11031 Taiwan, Republic of China; 6 Landseed Sports Medicine Center, Landseed International Hospital, No.77, Guangtai Rd., Pingzhen Dist., Taoyuan, 32449 Taiwan, Republic of China; 7 Department of Medical Education, Research and Quality Management, Landseed International Hospital, No.77, Guangtai Rd., Pingzhen Dist., Taoyuan, 32449 Taiwan, Republic of China; 8 Division of Health Care Management, Department of Community Medicine, Landseed International Hospital, No.77, Guangtai Rd., Pingzhen Dist., Taoyuan, 32449 Taiwan, Republic of China

**Keywords:** Health-related quality of life, Exercise, Physical activity, Cohort study

## Abstract

**Background:**

Physical activity is related to health-related quality of life, but little evidence from multiple waves of panel data in Asian countries area available. This study aims to explore the impacts of different degree of regular exercise on the trajectories of physical and mental dimensions of health-related quality of life (HRQOL) for community-dwelling Taiwanese adults during 2006–2014.

**Methods:**

Data were derived from the “Landseed Integrated Outreaching Neighborhood Screening (LIONS)” study, with 6182 adults enrolled at the baseline and subsequently followed up to three times till 2014. Linear mixed-effects modeling approach was employed to evaluate the growth curve models of HRQOL (with 16,281 observations) by linear & quadratic time effects, regular exercise (5-level moderate-intensity physical activity), and major influential factors of HRQOL.

**Results:**

Regular exercise showed significantly positive dose-response effects on physical HRQOL (β =1.27~2.54), and regular exercise of 150 min or more showed positive effects on mental HRQOL (β = 1.55~2.03). Besides, irregular exercise could also improve both physical and mental HRQOL (β = 1.27 & β = 0.87). However, such effects were not significant over time (at time slope) on HRQOL. In addition, physical and mental HRQOL improved across time (β = 1.01 and 1.49, respectively), but the time quadratic effect would significantly offset a little bit on physical dimension (β = − 0.22). Moreover, being female, increasing age, living alone, or poorer health status were related to lower physical HRQOL; and being younger, living alone, or poorer health status were associated with lower mental HRQOL.

**Conclusions:**

The positive dose-response relationship between regular exercise and HRQOL or its domains was demonstrated for community-dwelling Taiwanese adults. Thus, a regular exercise habit (better ≧150 min per week) is advised for community-based healthcare professionals and the government to incorporate into health promotion strategies and plans.

## Introduction

Numerous studies contend that regular exercise or health promoting or leisure time physical activity (PA) has a variety of health benefits. Some researchers assert that regular exercise can effectively lower the prevalence of many diseases [1–3], and postpone the onset of disabilities [4]. In addition, health promoting PA can also improve physical health [2], and mental health [5–11].

Health-related quality of life (HRQOL) has emerged as an important multidimensional concept in clinical and public health research in past decades [9, 12, 13], and been recognized as a desired health outcome for evaluating preventive or therapeutic health plans [14, 15]. The relationship between PA (including exercise) and HRQOL has been widely explored, and consistently demonstrated to be a positive association across age groups and countries [8, 16, 17]. Higher levels of PA were related to better HRQOL [18]. Higher dose of exercise exerted larger improvements in physical and mental quality-of-life [10].

Risk and protective factors have been found to be related to HRQOL. Older age, female gender, living alone, lower education, and poorer physical health (i.e., more diseases or medications) would be associated with worse HRQOL [19–21]. Moreover, unhealthy behaviors (i.e., smoking, alcohol drinking, betel-nut chewing) and mental/psychiatric disorders were related to poorer HRQOL [22–26].

Although the impacts of PA on HRQOL have received great attentions, there are research gaps in the literature. First, the vast majority of studies on the relations of PA to HRQOL have been conducted in Western countries, whereas fewer studies were carried out in Asian nations. Because of the differences in life style, healthcare, and social determinants between Western nations and non-Western nations, further replication of prior observations are warranted. Second, although past studies explored the effects of different intensity or time of PA on physical or mental health, most of the findings of positive association of PA with HRQOL were usually found in cross-sectional studies or in the only two-wave longitudinal studies [8, 11, 27–31]. The time effect of physical activities on HRQOL was little explored, and the time-varying covariates were not considered.

This study aims to explore the impacts of different degree of regular exercise on the trajectories of physical and mental dimensions of HRQOL for adults who participated in a multiple-wave community-based health screening program in Taiwan during 2006–2014.

## Methods

### Study design and sample population

Data were derived from a cohort study “Landseed Integrated Outreaching Neighborhood Screening (LIONS)” which has been conducted by the Landseed International Hospital in Taiwan to surveil chronic diseases (e.g., type 2 Diabetes mellitus, hypertension, cancers, cardiovascular diseases, osteoporosis, gout, psychiatric disorder) and relevant risk factors routinely for community-dwelling Taiwanese adults aged 30 or over. A sample was drawn by the probability-proportional-to-size sampling from the registered households of the Pingzhen district of Taoyuan City, Taiwan. The districts was chosen because it is the primary area of the patients cared by Landseed International Hospital from the LION project. They were invited to receive on-site health screening examinations to investigate four regulated cancers and other prevailing diseases, and a face-to-face survey interview. The design and implementation of LIONS was delineated in depth in a previous study [32].

LIONS was initiated in 2006 and enrolled 6182 individuals in nine admission cohorts during 2006–2014 (Table 1). All enrollees, with the free will to partake in any follow-up health screening, would be contacted 2 years after the baseline. Follow-ups were made for those admission cohorts at varying intervals, ranging from 1 to 3 years. Consequently, there were up to 4 waves for each admission cohort, and the observations by waves were 6182, 4268, 3424 and 2407 participants, respectively. In total, 16,281 observations were included in the analyses. By examining the goodness of fit, the sample was significantly different from the population: the sample was consisted of more females, older, and lower educated persons.

**Table 1:** Enrollment in health screening and subsequent participation in follow-ups

Admission cohorts	Year								
	2006	2007	2008	2009	2010	2011	2012	2013	2014
2006	1605^a^		1137^b^			874^c^			649^d^
2007		2068^a^		1483^b^		1155^c^			782^d^
2008			1424^a^		1066^b^	949^c^			654^d^
2009				676^a^		425^b^	321^c^		269^d^
2010					128^a^		63^b^	59^c^	53^d^
2011						156^a^		71^b^	66^c^
2012							56^a^		23^b^
2013								39^a^	
2014									30^a^

^a^Enrollment at baseline (*N* = 6182, across admission cohorts)

^b^1^st^ follow-up participation (*n* = 4268, across admission cohorts)

^c^2^nd^ follow-up participation (*n* = 3424, across admission cohorts)

^d^3^rd^ follow-up participation (*n* = 2407, across admission cohorts)

### Measures

#### The outcome variable

Health-related quality of life was measured by Taiwan version of Medical Outcomes Study short-form 36-item (SF-36) questionnaire [33], which was validated from the original SF-36 questionnaire [34, 35]. The SF-36 questionnaire evaluates eight constructs of generic health and well-being, including physical functioning, role limitations due to physical problems, bodily pain, general health, vitality, social functioning, role limitation due to emotional problems, and mental health [36]. Based on the scores of eight constructs, two overall scores of physical component summary (PCS) and mental component summary (MCS) were computed, ranging from 0 to 100 [37]. Higher PCS and MCS scores indicate better perceived physical and mental health, respectively. The Cronbach’s alpha of the 8 domains in Taiwanese population ranged from 0.76 to 0.92, except the internal consistency of social function was only 0.65 [33].

#### Health behaviors

Health behaviors involved several time-varying variables. The primary indicator, regular exercise habit, was measured according to 2008 Physical Activity Guidelines for Americans (PAGA) [38]. PAGA suggests physical activity level can be measured as four levels: inactive, low activity (<150 min), medium activity (150–299 min), and high activity (≧300 min). The participants were asked about the frequency of doing exercise and the time in minutes spent on exercise per week. The questions were “On average, how many times do you have the regular exercise per week, and how many minutes do you spend on doing the regular exercise per time?”, and “What kind of exercise do you regularly have?” (light-, moderate-, vigorous- or very vigorous-intensity exercise)”. In this study, we added another level, irregular exercise, by defining doing physical activities irregularly, and the average time spent on exercise was less than 10 min per week. In total five levels of exercise were defined (inactive, irregular exercise, low, medium, and high).

Other unhealthy behaviors, including current tobacco smoking and betel-nut chewing, were assessed by three binary indicators (1 = yes, 0 = no).

#### Health status

Mental status was measured by the 12-item Chinese Health Questionnaire (CHQ-12) to identify non-psychotic psychiatric disorders in community-dwelling Chinese people [39, 40]. The items were scored from “not at all” to “much more than usual”, and then recorded to 0 (no) and 1 (yes). A total score of CHQ-12, ranging from 0 to 12, was coded as 1 (i.e., score ≧4) to indicate a high risk for psychiatric disorder (otherwise as 0).

Morbidity was evaluated by the number of self-reported chronic diseases investigated at the health screening. The surveyed diseases include type 2 Diabetes mellitus (T2DM), hypertension, hyperlipidemia, kidney disease, cardiac disease, stroke, hepatic disease, gout, osteoporosis, asthma, psychiatric disease, nerve-related disease, intestinal disease, Tuberculosis and metabolic syndrome. A chronic disease was recognized when a participant self-reported to have such disease or take medications for curing the disease (1 = yes & 0 = no). Some clinical biomarkers were also applied to discover latent patients from those participants self-reporting without three diseases, including 1) hypertension recoded as 1, if systolic blood pressure ≧140 mmHg or diastolic blood pressure ≧90 mmHg; 2) T2DM recoded as 1, if fasting plasma glucose ≧126 mg/dL; and 3) hyperlipidemia recoded as 1, if total cholesterol (CHO) ≧240 mg/dL. The range of morbidity could be 0 to 15.

The use of medications was indexed by counting the number of investigated medications, including refreshing drugs, sleeping pills, sedative medicine, and painkillers (1 = yes & 0 = no for each medication). The score ranged from 0 to 4.

#### Social determinants

Demographic data, including age, gender and education, were incorporated in analytical models as time-constant variables. Age was assessed by the real age (in years) at baseline, and gender was coded as a binary measure. Education was indexed by the years of schooling education. Marital status was time-varying, which was re-classified into two groups: living with a spouse or no spouse.

#### Time-interval

This study was a multi-wave longitudinal design, and most predictors were time-varying. We calculated the time interval as the period (in years) between two adjacent participation dates (or the last day of the follow-up year for those enrollees not attending that follow-up screening). In addition, measures of time interval (i.e., Time and Time^2^) were also employed to explore the trajectory shape of HRQOL, specifically focusing on both physical and mental perspectives (i.e., PCS and MCS scores).

### Statistical analyses

Following a descriptive analysis, the linear mixed-effects modeling (LMM) approach was employed to fit multivariate growth curve models of PCS and MCS using SPSS version 21.0 [41]. The LMM method was chosen because it is suitable for dealing with repeated measures over time [42] and handling subjects with unbalanced design [43]. The percentages of missing data of all covariates for participants at four waves were less than 10%, ranged from 0 to 8.6% (see Additional file 1). The missing data were not under imputation for less the inconsequential [44]. The time-quadratic interaction terms were added in the models but all were insignificant; thus, those interaction terms were removed from the final models.

At last, only the intercepts and the linear time-slope parameters were retained. PCS and MCS scores were analyzed by three models. Model 0 only included the intercept and linear and quadratic time slopes as predictors to examine the proposed nonlinear growth curve model across time. Model 1 added the focused regular exercise and its interaction with linear time slope to capture total effects of regular exercise on individual changes of HRQOL over time. Finally, Model 2 included all other covariates and their interactions with linear time slope, in order to adjust the associations of regular exercise with HRQOL and to investigate their unique impacts on HRQOL.

## Results

Table 2 exhibits the characteristics of participants at first four waves of LIONS. In contrast to stable PCS scores ranging from 53.0 to 53.6, MCS scores steadily increased from 49.7 to 52.2 during 2006–2014. Of all 6182 enrollees, 54.7% were women, the average age was 53.5 years. Although the proportion of participants without doing exercise remained around 35.5 to 38.4%, the percentage of participants spending more than 150 min weekly on doing exercise increased obviously from 12.7 to 18.4%.

**Table 2:** Descriptive statistics of HRQOL and covariates at 4 waves

	Wave 1 (*N* = 6182)	Wave 2 (*N* = 4268)	Wave 3 (*N* = 3424)	Wave 4 (*N* = 2407)
PCS score^a^	53.0 ± 7.3	53.6 ± 6.8	53.4 ± 7.1	53.2 ± 7.1
MCS score^a^	49.7 ± 8.8	50.6 ± 8.3	50.9 ± 8.3	52.2 ± 8.1
Time-constant covariates				
Gender (female)^e^	54.7%			
Age (years)^a^	53.5 ± 13.1			
30–64 years old	80.2%			
≧65 years old	19.8%			
Education (years)^a^	10.1 ± 4.7			
Time-varying covariates				
Time (years)^a,b^	0 ± 0	2.1 ± 0.3	1.9 ± 0.9	2.9 ± 0.5
Marital status				
Living with the spouse	89.0%	89.4%	87.6%	87.6%
Living alone	11.0%	10.6%	12.4%	12.4%
Tobacco smoking (yes)	18.0%	14.0%	11.4%	11.0%
Betel-nut chewing (yes)	3.2%	2.0%	1.4%	1.0%
Psychiatric disorder (CHQ-12≧4)^e^	26.3%	21.6%	18.9%	17.2%
# of chronic diseases^a,c^	1.6 ± 1.4	1.7 ± 1.5	2.0 ± 1.5	2.0 ± 1.5
# of Medications^a,d^	0.1 ± 0.4	0.1 ± 0.3	0.1 ± 0.4	0.1 ± 0.4
Regular exercise status				
No exercise	36.9%	35.5%	38.4%	36.7%
Irregular exercise	40.0%	41.7%	37.8%	35.3%
<150 min	10.4%	10.0%	9.6%	9.5%
150–299 min	5.7%	5.9%	6.8%	8.1%
≧300 min	7.0%	6.9%	7.3%	10.3%

*Abbreviations*: *HRQOL* Health-related quality of life, *PCS* Physical component summary, *MCS* Mental component summary, *CHQ-12* 12-item Chinese Health Questionnaire, *T2DM* Type 2 Diabetes mellitus

^a^Mean ± SD (standard deviation)

^b^Time interval between two adjacent waves

^c^Self-reported and/or diagnosed chronic diseases, including T2DM, hypertension, hyperlipidemia, kidney disease, cardiac disease, stroke, hepatic disease, gout, osteoporosis, asthma, psychiatric disease, nerve-related disease, intestinal disease, Tuberculosis, and metabolic syndrome

^d^4 pills, including refreshing drugs, sleeping pills, sedative medicine, and painkiller

^e^The reference group: gender (male), psychiatric disorder (CHQ-12 = 0–3)

The characteristics of the participants according to their exercise level at the baseline were examined (see Table 3). There were differences in PCS and MCS across exercise levels, indicating a higher score accompanied with higher exercise level. The “no exercise” and “irregular exercise” groups were more likely to be female, older, lower educated, and having more chronic diseases.

**Table 3:** Similarity of HRQOL scores and covariates by regular exercise status at wave 1

*N* = 6182	Regular exercise status					*P*-value
	No exercise (*n* = 2141)	Irregular exercise (*n* = 2317)	<150 min (*n* = 601)	150–299 min (*n* = 332)	≧300 min (*n* = 405)	
PCS score^a,b^	52.3 ± 8.1	52.8 ± 7.3	54.7 ± 5.9	54.8 ± 5.1	54.5 ± 6.0	< 0.001
PF	86.8 ± 21.2	87.2 ± 19.1	93.1 ± 12.3	93.4 ± 11.9	92.8 ± 13.4	< 0.001
RP	79.7 ± 37.1	82.1 ± 35.0	86.6 ± 29.1	87.2 ± 28.4	88.2 ± 28.4	< 0.001
BP	81.8 ± 21.0	83.9 ± 18.9	83.7 ± 18.0	85.7 ± 16.8	85.3 ± 18.8	< 0.001
GH	62.0 ± 20.6	65.6 ± 19.8	68.1 ± 18.9	69.2 ± 17.6	70.3 ± 18.7	< 0.001
MCS score^a,b^	48.5 ± 9.3	50.2 ± 8.7	49.1 ± 8.2	50.9 ± 7.2	51.9 ± 8.2	< 0.001
VT	63.1 ± 18.8	67.7 ± 18.1	67.2 ± 16.2	70.7 ± 17.0	73.7 ± 17.7	< 0.001
SF	84.8 ± 18.4	86.7 ± 15.8	87.2 ± 14.1	89.2 ± 13.0	89.5 ± 14.5	< 0.001
RE	82.9 ± 34.9	85.6 ± 32.5	85.1 ± 31.3	89.1 ± 28.5	88.6 ± 28.0	0.001
MH	69.9 ± 17.5	73.1 ± 16.6	72.1 ± 16.0	74.4 ± 15.0	77.3 ± 16.9	< 0.001
Gender (female)^c^	56.1%	58.9%	42.9%	48.8%	45.9%	< 0.001
Age (years)^a,b,c^	51.1 ± 12.4	57.3 ± 13.6	46.2 ± 9.4	51.1 ± 10.0	55.8 ± 11.0	< 0.001
30–64 years old	85.3%	70.9%	96.7%	90.7%	80.2%	< 0.001
≧65 years old	14.7%	29.1%	3.3%	9.3%	19.8%	
Education (years)^a,b^	9.7 ± 4.6	9.4 ± 4.8	12.9 ± 3.3	11.8 ± 4.0	10.5 ± 4.1	< 0.001
Marital status^d^						
Living with the spouse	87.4%	90.0%	87.6%	91.8%	90.1%	0.016
Living alone	12.6%	10.0%	12.4%	8.2%	9.9%	
Tobacco smoking (yes)^c^	24.0%	12.3%	21.1%	15.4%	15.6%	< 0.001
Alcohol drinking (yes)^c^	13.0%	9.5%	19.5%	14.2%	13.5%	< 0.001
Betel-nut chewing (yes)^c^	5.1%	1.2%	5.3%	2.4%	2.2%	< 0.001
Psychiatric disorder (CHQ-12≧4)^c^	29.8%	25.3%	24.4%	22.5%	19.7%	< 0.001
# of chronic diseases^a,b,d^	1.64 ± 1.44	1.77 ± 1.51	1.32 ± 1.26	1.39 ± 1.28	1.58 ± 1.30	< 0.001
# of Medications^a,b,e^	0.11 ± 0.39	0.10 ± 0.36	0.08 ± 0.33	0.07 ± 0.29	0.10 ± 0.35	0.425

*Abbreviations*: *HRQOL* Health-related quality of life, *PCS* Physical component summary, *PF* Physical functioning, *RP* Role limitations due to physical problems, *BP* Bodily pain, *GH* General health, *MCS* Mental component summary, *VT* Vitality, *SF* Social functioning, *RE* Role emotional, *MH* Mental health, *CHQ-12* 12-item Chinese Health Questionnaire, *T2DM* Type 2 Diabetes mellitus

^a^ Mean ± SD (standard deviation); ^b^ by ANOVA; by ^c^ Chi-square test

^d^ Self-reported and/or diagnosed chronic diseases, including T2DM, hypertension, hyperlipidemia, kidney disease, cardiac disease, stroke, hepatic disease, gout, osteoporosis, asthma, psychiatric disease, nerve-related disease, intestinal disease, Tuberculosis, and metabolic syndrome

^e^ 4 pills, including refreshing drugs, sleeping pills, sedative medicine, and painkiller

Table 4 shows the trajectories of PCS & MCS with contemporary regular exercise and other covariates by LMM. For the trajectory of PCS, Model 0 indicated an increasing growth curve (β = 0.72, *p* < 0.001), but with decelerated growth rate (β = − 0.24, *p* < 0.001), of PCS score over time. After controlling for regular exercise and other covariates, the deceleration growth curve of PCS score across time was confirmed in Models 1 and 2, because of significantly positive linear time slope (β = 0.81, *p* < 0.001; β = 1.01, *p* < 0.05) but negative quadratic time slope (β = − 0.25 & -0.22, *p* < 0.001). Consequently, the trajectory of PCS score would increase at the beginning but slow down later on (i.e., a nonlinear, reversed U-shaped curve).

**Table 4:** Trajectories of HRQOL by regular exercise and covariates (*n* = 6182 persons, 16,281 observations)

Covariates	PCS score			MCS score		
	Model 0	Model 1	Model 2	Model 0	Model 1	Model 2
Fixed effects						
For intercept						
Intercept	53.02*** (52.83~53.20)	52.25*** (51.96~52.54)	60.67*** (59.26~62.07)	49.74*** (49.51~49.96)	48.52*** (48.17~48.87)	44.51*** (42.80~46.21)
Gender (female)^d^			−0.59** (− 0.99~ − 0.19)			− 0.26 (− 0.74~0.22)
Education (years)			0.03 (−0.01~0.08)			−0.002 (− 0.06~0.05)
AGE (years)			−0.13*** (− 0.15~ − 0.11)			0.09*** (0.07~0.11)
Time-varying covariates						
Living with the spouse^d^			0.62* (0.09~1.14)			1.77*** (1.14~2.40)
Tobacco smoking (yes)			0.14 (−0.36~0.64)			0.06 (− 0.54~0.67)
Betel-nut chewing (yes)			−0.18 (−1.20~0.83)			−0.32 (− 1.55~0.91)
Psychiatric disorder^d^			−2.93*** (−3.31~ − 2.54)			−6.17*** (−6.64~ − 5.71)
# of chronic diseases^b^			−0.71*** (− 0.83~ − 0.59)			0.09 (− 0.05~0.24)
# of Medications^c^			−2.22*** (− 2.68~ − 1.76)			− 2.42*** (− 2.97~ − 1.86)
Regular exercise status^d^						
≧300 min		2.27*** (1.57~2.97)	2.54*** (1.86~3.22)		3.39*** (2.55~4.24)	2.03*** (1.21~2.85)
150–299 min		2.68*** (1.91~3.45)	1.99*** (1.25~2.72)		2.52*** (1.59~3.44)	1.55** (0.65~2.44)
<150 min		2.39*** (1.78~3.00)	1.24*** (0.66~1.83)		0.71 (− 0.02~1.45)	0.65 (− 0.06~1.36)
Irregular exercise		0.55** (0.15~0.94)	1.27*** (0.89~1.66)		1.89*** (1.41~2.36)	0.87*** (0.40~1.33)
For time slope						
Time (years)^a^	0.72*** (0.42~1.03)	0.81*** (0.48~1.15)	1.01* (0.06~1.97)	0.79*** (0.42~1.17)	0.95*** (0.54~1.35)	1.49** (0.37~2.62)
Time^2^ (years)	−0.24*** (−0.35~ − 0.14)	− 0.25*** (− 0.36~ − 0.15)	−0.22*** (− 0.33~ − 0.10)	−0.06 (− 0.19~0.07)	−0.06 (− 0.19~0.07)	−0.05 (− 0.19~0.08)
Gender (female)^d^			0.02 (−0.23~0.27)			−0.14 (− 0.43~0.15)
Education (years)			−0.003 (− 0.03~0.03)			−0.01 (− 0.05~0.02)
AGE (years)			−0.01 (− 0.02~0.003)			−0.005 (− 0.02~0.01)
Time-varying covariates						
Living with the spouse^d^			−0.09 (−0.40~0.23)			−0.40* (− 0.77~ − 0.03)
Tobacco smoking (yes)			0.01 (−0.32~0.34)			−0.15 (− 0.54~0.24)
Betel-nut chewing (yes)			0.50 (−0.25~1.25)			0.69 (−0.21~1.59)
Psychiatric disorder^d^			0.11 (−0.13~0.35)			0.19 (−0.09~0.48)
# of 15 diseases^b^			0.02 (−0.05~0.10)			−0.08 (− 0.17~0.005)
# of Medications^c^			0.05 (−0.23~0.32)			0.10 (−0.22~0.43)
Regular exercise status^d^						
≧300 min		−0.02 (− 0.41~0.37)	0.03 (− 0.36~0.41)		−0.43 (− 0.89~0.04)	0.001 (− 0.46~0.47)
150–299 min		−0.24 (− 0.67~0.18)	0.08 (− 0.34~0.50)		−0.50 (−1.01~ − 0.001)	−0.11 (− 0.62~0.39)
<150 min		− 0.43* (− 0.78~ − 0.08)	0.03 (−0.32~0.38)		−0.10 (− 0.53~0.32)	−0.12 (− 0.54~0.30)
Irregular exercise		− 0.03 (− 0.26~0.20)	0.04 (− 0.19~0.28)		−0.21 (− 0.48~0.07)	0.07 (− 0.20~0.35)
Random effects						
Variance (Wave 1)	53.76***	53.01***	44.84***	78.02***	77.07***	65.81***
Variance (Wave 2)	29.31***	29.39***	27.81***	50.18***	50.46***	48.30***
Variance (Wave 3)	35.20***	35.03***	32.82***	51.28***	50.76***	47.63***
Variance (Wave 4)	23.73***	23.85***	26.37***	34.90***	35.25***	37.01***
Variance (Time)	4.25***	4.06***	2.59***	4.60***	4.44***	2.80***
-2LL	103,650.8	102,351.7	94,864.7	109,273.9	107,958.8	100,337.1
AIC	103,660.8	102,361.7	94,874.7	109,283.9	107,968.8	100,347.1
BIC	103,699.0	102,399.9	94,912.6	109,322.1	108,007.0	100,385.0

*Abbreviations*: *HRQOL* Health-related quality of life, *PCS* Physical component summary, *MCS* Mental component summary, *T2DM* type 2 Diabetes mellitus, *−2LL* −2 restricted log-likelihood, *AIC* Akaike’s information criterion, *BIC* Schwarz’s Bayesian criterion

*p < 0.05; ** *p* < 0.01; *** *p* < 0.001

^a^Time interval between two adjacent waves

^b^Self-reported and/or diagnosed chronic diseases, including T2DM, hypertension, hyperlipidemia, kidney disease, cardiac disease, stroke, hepatic disease, gout, osteoporosis, asthma, psychiatric disease, nerve-related disease, intestinal disease, Tuberculosis, and metabolic syndrome

^c^4 pills, including refreshing drugs, sleeping pills, sedative medicine, and painkiller

^d^The reference group: gender (male), marital status (living alone), psychiatric disorder (CHQ-12<4), regular exercise status (no exercise)

Model 1 exhibited that doing exercise regularly had significantly positive effects on the intercept of PCS score, including those irregular PA. That means those participants who were doing exercise at the beginning had better physical at the beginning. For the effects of regular exercise on the time slope, only doing exercise 150 min weekly would reduce the PCS score over time (β = − 0.43, *p* < 0.05). With the inclusion of other covariates in Model 2, regular exercise still had significantly positive effects on the initial status of PCS score (at the intercepts), which seemed to be a dose-response relationship based on the levels of effective PA (β = 1.20–2.55, *p* < 0.001). However, regular exercise became insignificant for the changes of PCS score over time (*p* > 0.05), indicating constant effects of regular exercise on PCS score across time.

Model 2 also showed that some covariates had substantial and varying effects on the intercept, but not on the time slope, of PCS score. For time-constant variables, female (β = − 0.59, *p* < 0.01) or older β = − 0.13, *p* < 0.001) participants had a stably lower initial PCS score over time. Similarly, there were five time-varying covariates with significant effects only on the intercept of PCS score. Both living with a spouse (β = 0.62, *p* < 0.05) was positively related to the initial status of PCS score. Psychiatric disorder (β = − 2.93, *p* < 0.001), the number of chronic diseases (β = − 0.71, *p* < 0.001) and taken medications (β = − 2.22, *p* < 0.001) had negative effects on the intercept of PCS score. However, these covariates were in-significant across time.

For the trajectory of MCS score, Model 0 showed an increasing growth curve (β = 0.79, *p* < 0.001) across time. Even if regular exercise and other covariates were controlled in Models 1 and 2, the increasing tendency of MCS score over time still existed, due to significantly positive linear time slope (β = 0.95, *p* < 0.001; β = 1.49, *p* < 0.01). That means the growth curve of MCS score would be an increasing linear trajectory over time.

Model 1 indicated that regular exercise had significantly positive and varying impacts on the intercept of MCS score (*p* < 0.001), except for doing low level exercise (< 150 min weekly). However, doing exercise regularly had no time effect on MCS score in Model 1.

After controlling for other covariates in Model 2, most levels of regular exercise, except for the low level (<150 min per week), were significantly and positively associated with MCS score at the intercept. Additionally, for those significant regular exercise, higher level of effective PA (i.e., ≧300 min weekly) was related to better initial status of MCS score (β = 2.03, *p* < 0.001). The effect of irregular physical activities was still significant. However, regular exercise had no significant effect on changing the MCS score over time.

Model 2 exhibited that several covariates had significant and varying impacts on the intercept and linear time slope of MCS score. Age (β = 0.09, *p* < 0.001) was positively related to the initial status of MCS score, indicating that older participants would perceive better mental QOL constantly. Living with a spouse had a positive effect on the intercept (β = 1.77, *p* < 0.001) but a negative impact on the time slope (β = − 0.40, *p* < 0.05) of MCS score. These results revealed that participants living with a spouse had higher MCS score but it would decrease over time. Both psychiatric disorder (β = − 6.17, *p* < 0.001) and the number of taken medications (β = − 2.42, *p* < 0.001) only had negative effects on the initial status of MCS score. Namely, participants with the psychiatric disorder or taking more drugs would have lower MCS score at the very beginning. However, morbidities and medications were not significantly on the time slope.

## Discussion

This study used a multiple-wave longitudinal panel data of community-dwelling adults in Northern Taiwan to examine the effects of regular exercise on health-related quality of life over time among community adults. Those with regular exercise showed a significant improving effect on physical health than those without doing exercise at the intercepts, especially if the regular exercise time was more than 150 min a week. Regular exercise also showed positive effects (at the intercepts) on mental health if the exercise time was 150 min or more in a week. However, the regular exercise effects were not significant over time (at time slope) on either physical or mental health scores. In addition, physical health and mental health improved over time, but the time effect would offset a little bit on physical health. Moreover, being female, increasing age, living alone, or poorer health status were related to lower physical health, but drinking alcohol was related to better physical health. The participants who were younger, lived alone or had poorer health status would have worse mental health.

The present research demonstrates that compared to non-exercising almost all levels of PA had positive effects on PCS and MCS. These results were consistent with the literature that usually speculated the varying positive associations of exercise/PA with overall quality-of-life [16, 18, 45, 46], PCS [10, 30, 46–50], MCS [10, 11, 30, 47], or subjective well-being [27, 28, 51, 52].

In our study, 76.9% of our participants did not have exercise habits or did not meet the recommendation criteria. Previous research indicated that 86% of Taiwanese did not meet the national recommendation [28]. However, the present research showed that even irregular levels of PA lower than currently recommended for beneficial health effects [38] were also related to the improvement in PCS. Previous research suggested the positive association of light-intensity PA to different aspects of well-being [28], and the low PA level (40–179 min per week) [47] or even irregular excise [46] still had significantly positive effects on PCS. Therefore, doing exercise regularly would be beneficial for improving physical health, no matter the amount and intensity of PA. Further, this study found the insignificant impact of PA on the linear time slope, indicating that the effects of PA on HRQOL would be constant over time. A curvilinear trend between PA levels and HRQOL, particularly physical functioning and vitality in previous study [47], was not discovered in this study. It is possibly attributed to using different measures of PA, or missing some confounders to physical health.

Regarding to the PA effect on mental health, although the level of <150 min was insignificant, further analyses of MCS subscales (see Additional file 2) revealed that it had significantly positive effects on three domains, including vitality, social functioning and mental health. Wicker and Frick [27] suggested that light-intensity PA would be beneficial for improving individuals’ mental health too. However, some past research found the insignificant relationship between PA and role limitations due to emotional problems [11, 50]. Therefore, all levels of PA would have varying influences on MCS and its domains, including irregular exercise. Besides, our results support the conclusion that changes in HRQOL were dose dependent on the amount of PA [10, 18, 46–49].

The trajectory of physical health would increase at first and decrease after a period of time, consistent with the literature. Previous studies have found the trajectories of HRQOL for chronic diseases may be improved by medical interventions [53–55]. Moreover, this study found the aging effect would offset the physical health in quality-of-life, consistent with previous research [56]. The trajectory of mental health was also found to be positively related to time linear effect as previous research [57, 58]. The time effect to improve mental health can be explained by the socioemotional selectivity theory [59]. Increasing age will limit the future prospections of people and then make them focusing on positive information and seeking for the emotional satisfaction [60, 61], and thus people would employ various coping strategies to focus on potential positive outcomes of unfavorable events such as chronic diseases or disabilities.

Previous studies usually contended that women had worse HRQOL than men in general or in specific domains, even after controlling for sociodemographic characteristics, economic status and chronic diseases [20, 62–64]. However, our results indicated that female adults only had significantly lower PCS score. The insignificant gender difference in MCS score could be attributed to the inclusion of psychiatric/mental disorder and PA/exercise in the models, which would reduce the direct effect of gender on HRQOL, especially MCS. Previous studies concluded that psychiatric disorder (e.g., anxiety, depression) was more prevalent in women and most important predictor of MCS score [25], and PA had larger positive effects on MCS for women [30, 65]. This study discovered that chronic diseases had negative impacts on PCS score, consistent with previous literature. However, the relationship between chronic diseases and MCS score was found to be nonlinear, which could be partially explained by psychological adjustment to chronic diseases [59, 60]. Namely, the positivity effect of psychological adjustment could be offset by the cumulative burden of chronic diseases over time, resulting in the curvilinear association of MCS score with chronic diseases. Additionally, only one unhealthy behavior (i.e., alcohol drinking) was positively related to PCS score, which was supported by some studies [24, 66] but contrary to other research [23]; nevertheless, other unhealthy behaviors had insignificant and inconsistent impacts on HRQOL. These results could be attributed to binary non-quantity measures [67] and small cases with risky behaviors (e.g., betel-nut chewing).

### Study limitations

This study could be improved in several ways. First, although this study was based on a sizable sample with four repeated observations during 2006–2014, the sample could not be generalized to the district or other populations. Further replications with data collected in Taiwan and other Asian countries would be required to extend the generalizability of our findings. Second, self-reported exercise data could be refined by the adoption of objective instruments, which may reduce measurement errors (i.e., potential overlapping between self-reported data and subjective HRQOL) and avoid incorrect statistical inference [18, 28, 52]. Third, the intensity of regular exercise and all the forms of physical activity can be measured by metabolic equivalents (METS) for a better measure of physical activity. Fourth, a refined model with fewer significant covariates but more interaction terms of PA and time could generate more accurate effects of PA on the trajectory of HRQOL over time. Fourth, alcohol drinking behavior was not included in the model, because the amount of alcohol drinking was not available. Alcohol drinking may be a confounder to HRQOL. In fact, we tried to put alcohol drinking (yes/no) in the models, and it showed a positive effect on PCS at the intercept but non-significant on the time slope or on MCS. A more detailed alcohol intake is suggested to examine the longitudinal effect of alcohol drinking on HRQOL. Fifth, there were attritions in the follow-ups. Only 1.5%~ 4.2% of the non-participants because of death or loss in the follow-ups. Most of the non-participants (more than 95%) did receive the invitation in the next following waves and considered or agreed to participate in the survey, but eventually they did not show up. We further compared the baseline characteristics between the participants and non-participants, and the participants were more likely to be healthier than the non-participants (please see Additional file 3). Therefore, the sample may be over-estimated the health and healthy lifestyle from the population.

## Conclusions

This study found the positive effects of doing regular exercise on HRQOL to the general population in Taiwan, and the dose-response relationship of regular exercise on HRQOL was found. Regular exercise improves physical and mental dimensions of HRQOL if the cumulative time of exercise is 150 min or more per week. Even though an irregular exercise (less than 150 min a week) would be beneficial for perceived physical health. We suggest that a regular exercise habit should be built up and promoted by community-based healthcare organizations and the government, and cumulating the exercise time for at least 150 min per week is even better. We also suggest that future research should identify the long-term effects of various types, intensities and time amount of PA via objective measurement on various domains of HRQOL, as they could be critical to the design of effective and specific health promotion plans for improving HRQOL of community-dwelling people.

## Supplementary information

**Additional file 1.** Missing data information of HRQOL and covariates at 4 waves.

**Additional file 2. **Trajectories of HRQOL (i.e., mental scores) by regular exercise and covariates (*n* = 6182 persons, 16,281 observations).

**Additional file 3.** Comparisons between non-participation and participation at Wave2 – Wave4 by predictors at baseline.

## Data Availability

The data that support the findings of this study are available from the Landseed International Hospital but restrictions apply to the availability of these data, which were used under license/permission for the current study, and so are not publicly available. However, data are available with permission of the Landseed International Hospital, but can only be accessed in the Landseed International Hospital (currently without the virtual access option).

## Abbreviations

CHO: Total cholesterol
CHQ-12: 12-item Chinese Health Questionnaire
HRQOL: Health-related quality of life
LIONS: Landseed Integrated Outreaching Neighborhood Screening
MCS: Mental component summary
METS: Metabolic equivalents
PA: Physical activity
PCS: Physical component summary
QOL: Quality of life
SF-36: Medical Outcomes Study short-form 36-item
T2DM: Type 2 Diabetes mellitus
