# Supplementary material for: Regular exercise and the trajectory of health-related quality of life among Taiwanese adults: a cohort study analysis 2006–2014
Source: BMC Public Health. 2019 Oct 23;19:1352. doi: 10.1186/s12889-019-7662-8 (PMC6806516; doi:10.1186/s12889-019-7662-8)
Supplement: Supplementary file 1 — Additional file 1. Missing data information of HRQOL and covariates at 4 waves. [file 12889_2019_7662_MOESM1_ESM.pdf]

Additional file 1 Missing data information of HRQOL and covariates at 4 waves

	Wave 1	Wave 2	Wave 3	Wave 4
	**(N = 6182)**	**(N = 4268)**	**(N = 3424)**	**(N = 2407)**
PCS score*	53.0±7.3	53.6±6.8	53.4±7.1	53.2±7.1
Missing (%)	8.6	4.8	1.9	0.9
MCS score*	49.7±8.8	50.6±8.3	50.9±8.3	52.2±8.1
Missing (%)	8.6	4.8	1.9	0.9
Time-constant covariates				
Gender				
Female (%)	54.7			
Male (%)	45.3			
Missing (%)	0.0			
Age* (years)	53.5±13.1			
30-64 years (%)	80.2			
≧65 years (%)	19.8			
Missing (%)	0.0			
Education (years)*	10.1±4.7			
Missing (%)	1.6			
Time-varying covariates				
Time (years)*,§	0±0	2.1±0.3	1.9±0.9	2.9±0.5
Marital status				
Lived with a spouse (%)	84.7	88.4	86.5	86.2
Lived alone (%)	10.5	10.4	12.3	12.2
Missing (%)	4.8	1.2	1.3	1.6
Tobacco smoking (yes)				
No (%)	78.5	85.4	87.8	88.8
Yes (%)	17.2	13.9	11.3	10.9
Missing (%)	4.3	0.7	0.8	0.2
Alcohol drinking (yes)				
No (%)	83.4	89.9	93.3	94.3
Yes (%)	11.8	8.1	5.1	5.0
Missing (%)	4.8	2.0	1.6	0.7
Betel-nut chewing (yes)				

No (%)	92.5	97.3	97.8	98.7
Yes (%)	3.1	2.0	1.4	1.0
Missing (%)	4.5	0.8	0.8	0.3
Psychiatric disorder				
CHQ-12＜4 (%)	69.2	75.7	79.5	81.8
CHQ-12≧4 (%)	24.7	20.8	18.5	17.0
Missing (%)	6.1	3.5	2.0	1.2
# of chronic diseases[*,†]	1.6±1.4	1.7±1.5	2.0±1.5	2.0±1.5
Missing (%)	0.0	0.0	0.0	0.0
# of Medications[*,‡]	0.1±0.4	0.1±0.3	0.1±0.4	0.1±0.4
Missing (%)	6.5	3.8	1.1	0.4
Regular exercise status				
No exercise (%)	34.6	34.2	37.8	36.6
Ineffective exercise (%)	37.5	40.3	37.2	35.1
＜150 minutes (%)	9.7	9.6	9.5	9.5
150-299 minutes (%)	5.4	5.7	6.7	8.1
≧300 minutes (%)	6.6	6.7	7.2	10.2
Missing (%)	6.2	3.4	1.6	0.5

[*]Mean±SD (standard deviation)

[§]Time interval between two adjacent waves

[†]Self-reported and/or diagnosed chronic diseases, including T2DM, hypertension, hyperlipidemia, kidney disease, cardiac disease, stroke, hepatic disease, gout, osteoporosis, asthma, psychiatric disease, nerve-related disease, intestinal disease, Tuberculosis, and metabolic syndrome

[‡]4 pills, including refreshing drugs, sleeping pills, sedative medicine, and painkiller

Abbreviations: HRQOL, health-related quality of life; PCS, physical component summary; MCS, mental component summary; CHQ-12, 12-item Chinese Health Questionnaire; T2DM, type 2 Diabetes mellitus
