# Supplementary material for: Regular exercise and the trajectory of health-related quality of life among Taiwanese adults: a cohort study analysis 2006–2014
Source: BMC Public Health. 2019 Oct 23;19:1352. doi: 10.1186/s12889-019-7662-8 (PMC6806516; doi:10.1186/s12889-019-7662-8)
Supplement: Supplementary file 2 — Additional file 2. Trajectories of HRQOL (i.e., mental scores) by regular exercise and covariates (n = 6182 persons, 16,281 observations). [file 12889_2019_7662_MOESM2_ESM.pdf]

Additional file 2 Trajectories of HRQOL (i.e., mental scores) by regular exercise and covariates (n = 6182 persons, 16281 observations)

Covariates	Mental scores				
	**VT**	**SF**	**RE**	**MH**	**MCS**
Fixed effects	(n=5961)	(n=5956)	(n=5960)	(n=5960)	(n=5922)
For intercept					
Intercept	59.75***	88.54***	86.89***	66.05***	44.51***
	(56.18~63.32)	(85.27~91.81)	(80.40~93.39)	(62.80~69.30)	(42.80~46.21)
Gender (female)[¶]	-0.91	-0.50	-0.02	-1.17*	-0.26
	(-1.92~0.10)	(-1.42~0.43)	(-1.86~1.82)	(-2.09~-0.25)	(-0.74~0.22)
Education (years)	0.06	-0.07	0.04	0.01	-0.002
	(-0.06~0.18)	(-0.17~0.04)	(-0.17~0.25)	(-0.10~0.11)	(-0.06~0.05)
AGE (years)	0.09***	-0.03	-0.03	0.12***	0.09***
	(0.05~0.14)	(-0.07~0.01)	(-0.11~0.05)	(0.08~0.16)	(0.07~0.11)
Time-varying covariates					
Living with the spouse[¶]	3.19***	2.96***	5.33***	2.89***	1.77***
	(1.86~4.53)	(1.74~4.17)	(2.91~7.75)	(1.68~4.11)	(1.14~2.40)
Tobacco smoking (yes)	0.38	0.09	-1.54	0.77	0.06
	(-0.89~1.66)	(-1.07~1.26)	(-3.86~0.77)	(-0.39~1.93)	(-0.54~0.67)
Betel-nut chewing (yes)	1.12	-1.52	-3.22	0.97	-0.32
	(-1.46~3.70)	(-3.88~0.85)	(-7.92~1.48)	(-1.37~3.32)	(-1.55~0.91)
Psychiatric disorder[¶]	-11.19***	-7.95***	-16.71***	-12.11***	-6.17***
	(-12.17~-10.22)	(-8.84~-7.06)	(-18.49~-14.93)	(-13.00~-11.23)	(-6.64~-5.71)
# of chronic diseases[†]	-0.28	-0.33*	-0.71*	-0.05	0.09
	(-0.59~0.03)	(-0.61~-0.04)	(-1.27~-0.15)	(-0.33~0.23)	(-0.05~0.24)

# of Medications[‡]	-4.76***	-4.84***	-6.52***	-4.83***	-2.42***
	(-5.93~-3.59)	(-5.90~-3.77)	(-8.64~-4.39)	(-5.90~-3.77)	(-2.97~-1.86)
Regular exercise status[¶]					
≧300 minutes	8.97***	3.16***	4.08*	5.04***	2.03***
	(7.24~10.70)	(1.58~4.74)	(0.94~7.22)	(3.47~6.62)	(1.21~2.85)
150-299 minutes	6.12***	3.57***	4.02*	2.89**	1.55**
	(4.24~8.00)	(1.87~5.27)	(0.62~7.42)	(1.18~4.61)	(0.65~2.44)
＜150 minutes	3.62***	1.89**	0.76	1.73*	0.65
	(2.14~5.11)	(0.53~3.25)	(-1.94~3.47)	(0.38~3.09)	(-0.06~1.36)
Ineffective exercise	3.85***	1.53**	2.26*	2.04***	0.87***
	(2.86~4.83)	(0.63~2.43)	(0.47~4.05)	(1.15~2.94)	(0.40~1.33)
For time slope					
Time (years) [§]	3.32**	3.00**	8.04***	1.15	1.49**
	(0.87~5.77)	(0.89~5.10)	(3.97~12.11)	(-1.06~3.36)	(0.37~2.62)
$Time^2$ (years)	-0.12	-0.17	-0.66*	-0.12	-0.05
	(-0.41~0.18)	(-0.43~0.09)	(-1.17~-0.16)	(-0.39~0.14)	(-0.19~0.08)
Gender (female)[¶]	-0.06	0.16	-0.87	-0.38	-0.14
	(-0.70~0.58)	(-0.39~0.70)	(-1.93~0.18)	(-0.95~0.20)	(-0.43~0.15)
Education (years)	-0.07	-0.01	-0.09	0.02	-0.01
	(-0.14~0.01)	(-0.08~0.05)	(-0.22~0.03)	(-0.05~0.09)	(-0.05~0.02)
AGE (years)	-0.02	-0.02	-0.04	0.004	-0.005
	(-0.04~0.01)	(-0.05~0.003)	(-0.09~0.01)	(-0.02~0.03)	(-0.02~0.01)
Time-varying covariates					
Living with the spouse[¶]	-0.79	-0.38	-1.47*	-0.44	-0.40*
	(-1.59~0.02)	(-1.09~0.32)	(-2.84~-0.11)	(-1.17~0.29)	(-0.77~-0.03)

Tobacco smoking (yes)	-0.34	-0.01	0.22	-0.49	-0.15
	(-1.19~0.51)	(-0.74~0.72)	(-1.20~1.63)	(-1.26~0.27)	(-0.54~0.24)
Betel-nut chewing (yes)	1.06	1.64	1.35	1.59	0.69
	(-0.90~3.01)	(-0.07~3.34)	(-1.96~4.66)	(-0.17~3.36)	(-0.21~1.59)
Psychiatric disorder¶	0.01	0.03	0.27	0.60*	0.19
	(-0.61~0.62)	(-0.51~0.57)	(-0.78~1.32)	(0.04~1.16)	(-0.09~0.48)
# of 15 diseases†	-0.07	-0.12	-0.26	-0.07	-0.08
	(-0.26~0.11)	(-0.28~0.04)	(-0.57~0.06)	(-0.24~0.10)	(-0.17~0.005)
# of Medications‡	0.15	0.51	-0.02	-0.03	0.10
	(-0.55~0.86)	(-0.11~1.13)	(-1.23~1.19)	(-0.67~0.61)	(-0.22~0.43)
Regular exercise status¶					
≧300 minutes	0.07	0.12	-0.61	-0.07	0.001
	(-0.94~1.08)	(-0.76~1.01)	(-2.33~1.11)	(-0.98~0.84)	(-0.46~0.47)
150-299 minutes	-0.03	-0.64	-0.18	-0.003	-0.11
	(-1.12~1.05)	(-1.59~0.32)	(-2.04~1.69)	(-0.99~0.98)	(-0.62~0.39)
＜150 minutes	-0.17	-0.92*	-0.13	-0.06	-0.12
	(-1.08~0.74)	(-1.72~-0.12)	(-1.69~1.43)	(-0.88~0.76)	(-0.54~0.30)
Ineffective exercise	0.23	-0.05	0.17	0.12	0.07
	(-0.37~0.83)	(-0.57~0.48)	(-0.86~1.19)	(-0.42~0.66)	(-0.20~0.35)
Random effects					
Variance (Wave 1)	294.5***	249.0***	989.8***	243.5***	65.81***
Variance (Wave 2)	223.5***	200.5***	750.9***	186.1***	48.30***
Variance (Wave 3)	227.6***	173.7***	740.6***	188.0***	47.63***
Variance (Wave 4)	190.5***	152.8***	579.0***	155.6***	37.01***
Variance (Time)	15.9***	6.4***	16.6***	12.1***	2.80***

-2LL	124994.8	120934.8	141107.9	121958.0	100337.1
AIC	125004.8	120944.8	141117.9	121968.0	100347.1
BIC	125042.8	120982.7	141155.9	122006.0	100385.0

**p* <0.05; ***p* <0.01; ****p* <0.001

§Time interval between two adjacent waves

†Self-reported and/or diagnosed chronic diseases, including T2DM, hypertension, hyperlipidemia, kidney disease, cardiac disease, stroke, hepatic disease, gout, osteoporosis, asthma, psychiatric disease, nerve-related disease, intestinal disease, Tuberculosis, and metabolic syndrome

‡4 pills, including refreshing drugs, sleeping pills, sedative medicine, and painkiller

¶The reference group: gender (male), age (≧65 years old), marital status (living alone), psychiatric disorder (CHQ-12＜4), exercise status (no exercise)

Abbreviations: HRQOL, health-related quality of life; VT, vitality; SF, social functioning; RE, role emotional; MH, mental health; MCS, mental component summary; T2DM, type 2 Diabetes mellitus; -2LL, -2 restricted log-likelihood; AIC, Akaike's information criterion; BIC, Schwarz's Bayesian criterion
