# Supplementary material for: Regular exercise and the trajectory of health-related quality of life among Taiwanese adults: a cohort study analysis 2006–2014
Source: BMC Public Health. 2019 Oct 23;19:1352. doi: 10.1186/s12889-019-7662-8 (PMC6806516; doi:10.1186/s12889-019-7662-8)
Supplement: Supplementary file 3 — Additional file 3. Comparisons between non-participation and participation at Wave2 – Wave4 by predictors at baseline. [file 12889_2019_7662_MOESM3_ESM.pdf]

Additional file 3 Comparisons between non-participation and participation at Wave2 – Wave4 by predictors at baseline

**N = 6,182**	**Wave 2 (N = 6,154)**			**Wave 3 (N = 6,110)**			**Wave 4 (N = 6,022)**		
	**non-Part. (n=1,886)**	**Part. (n=4,268)**	***p*-value**	**non-Part. (n=2,686)**	**Part. (n=3,424)**	***p*-value**	**non-Part. (n=3,615)**	**Part. (n=2,407)**	***p*-value**
**Covariates**	**%/Mean**	**%/Mean**		**%/Mean**	**%/Mean**		**%/Mean**	**%/Mean**	
Gender									
Female	53.6%	55.4%	0.188	52.6%	56.4%	0.003	53.7%	56.3%	0.047
Male	46.4%	44.6%		47.4%	43.6%		46.3%	43.7%	
Age (years)	54.99	52.82	<0.001	54.75	52.46	<0.001	54.00	52.68	<0.001
30 ~ 64	75.0%	82.5%	<0.001	75.3%	84.1%	<0.001	77.8%	83.9%	<0.001
≧65	25.0%	17.5%		24.7%	15.9%		22.2%	16.1%	
Education (years)	9.24	10.44	<0.001	9.61	10.48	<0.001	9.88	10.44	<0.001
No schooling	13.1%	6.4%	<0.001	11.4%	6.0%	<0.001	10.2%	5.5%	<0.001
below Senior HS	41.1%	38.0%		39.7%	38.1%		38.9%	38.5%	
Senior HS	27.7%	32.1%		28.5%	32.7%		28.5%	34.6%	
above Senior HS	18.1%	23.5%		20.5%	23.2%		22.4%	21.5%	
Marital status									
Living with spouse	85.7%	90.4%	<0.001	86.9%	90.5%	<0.001	87.4%	91.0%	<0.001
Single	14.3%	9.6%		13.1%	9.5%		12.6%	9.0%	
Tobacco smoking (Yes)	21.6%	16.4%	<0.001	21.1%	15.5%	<0.001	19.5%	15.6%	<0.001
Betel-nuts chewing (Yes)	4.7%	2.5%	<0.001	4.2%	2.4%	<0.001	3.9%	2.1%	<0.001
Psychiatric status (by CHQ-12)	3.05	2.95	0.040	2.98	2.99	0.825	2.97	3.00	0.462

Normal	70.4%	75.2%	<0.001	73.3%	74.0%	0.563	73.4%	74.3%	0.421
Psychiatric disorder*	29.6%	24.8%		26.7%	26.0%		26.6%	25.7%	
# of 15 diseases†	1.79	1.54	<0.001	1.77	1.49	<0.001	1.72	1.45	<0.001
# of 4 Pills taken‡	0.09	0.10	0.669	0.10	0.10	0.669	0.10	0.09	0.667
Regular exercise status									
No exercise	43.1%	34.3%	<0.001	41.6%	33.3%	<0.001	39.5%	33.0%	<0.001
Irregular exercise	37.8%	40.9%		39.3%	40.6%		39.6%	40.7%	
＜150 minutes	8.2%	11.2%		8.9%	11.5%		9.0%	12.4%	
150~299 minutes	5.0%	6.1%		4.7%	6.6%		5.3%	6.4%	
≧300 minutes	5.8%	7.5%		5.6%	8.1%		6.6%	7.6%	
PCS score	52.25	53.38	<0.001	52.41	53.52	<0.001	52.69	53.56	<0.001
MCS score	49.52	49.70	0.492	49.67	49.64	0.920	49.45	50.00	0.024
Attrition									
Died	21			48			88		
Lost to follow-up	7			24			72		

* CHQ-12 score≧4

† 15 self-reported and/or diagnosed chronic diseases, including T2DM, hypertension, hyperlipidemia, kidney disease, cardiac disease, stroke, hepatic disease, gout, osteoporosis, asthma, psychiatric disease, nerve-related disease, intestinal disease, Tuberculosis, and metabolic syndrome

‡ 4 pills, including refreshing drugs, sleeping pills, sedative medicine, and painkiller

Abbreviations: PCS, physical component summary; MCS, mental component summary; T2DM, type 2 Diabetes mellitus; non-Part., non-Participation; Part., Participation
